# Mosaic Darier's Disease: A Case of Unilateral Localized Type I Segmental Darier's Disease

**DOI:** 10.1002/ccr3.70306

**Published:** 2025-03-05

**Authors:** Lina Al‐Soufi, Aya Marashli, Hamza Warda, Zuheir Al‐Shehabi

**Affiliations:** ^1^ Department of Dermatology National Hospital Latakia Syria; ^2^ Faculty of Medicine Al‐Baath University Homs Syria; ^3^ Faculty of Medicine Tishreen University Latakia Syria

**Keywords:** case report, Darier‐white disease, genetic, mosaicism, skin diseases

## Abstract

Darier's disease (DD), also known as Darier‐White disease, is a rare autosomal dominant genodermatosis resulting from mutations in the ATP2A2 gene. It commonly manifests as keratotic papules or plaques in seborrheic areas and is often triggered by heat, sweating, and trauma. A rare mosaic variant, segmental Darier's disease, occurs due to post‐zygotic mutations and presents along Blaschko's lines. We describe a 41‐year‐old male presenting with pruritic, hyperpigmented, keratotic papules distributed unilaterally on the left trunk and upper thigh. The lesions, following Blaschko's lines, worsened with heat. There was no family history of similar conditions. Histopathology revealed classic features of Darier's disease, including irregular acantholysis, dyskeratosis with corps ronds and grains, and suprabasal acantholysis, confirming the diagnosis of type I segmental Darier's disease. Treatment involved corticosteroids twice daily, salicylic acid 2% gel for hyperkeratosis, and lifestyle modifications. Isotretinoin was avoided due to fertility plans. This case highlights the distinctive presentation of type I segmental Darier's disease and underscores the importance of accurate diagnosis through clinical and histopathological correlation. Increased awareness of this rare condition is crucial for timely diagnosis, effective symptom management, and tailored treatment strategies.

**Summary**

- This case highlights distinctive features of the rarely presenting type I segmental Darier's disease and emphasizes its atypical characteristics.
- Increased clinical awareness of these features will help ensure precise diagnosis for effective patient management and counseling.

## 1 Introduction

Darier's disease, also known as Darier‐White disease and follicular keratosis, was originally described in 1889 by the two dermatologists Darier and White [1]. It is a rare skin disorder that is inherited dominantly. The severity of the condition can vary widely among those affected [2]. This pathological condition results from either spontaneous or inherited mutations in the gene encoding the calcium‐dependent ATPase of the endoplasmic reticulum, known as the SERCA2 (sarcoplasmic/endoplasmic reticulum Ca (2^+^) ATPase) protein, which is mapped to the 12q23‐q24 subregions of chromosome 12 [1]. The frequency of Darier's disease is between 1 in 55,000 and 1 in 100,000 within the population, with an equal incidence rate among both genders. The first signs of the disease usually appear in people between the ages of 6 and 20 [3].

The disease is characterized by keratotic papules or plaques in the seborrheic areas with occasional nail or mucosal involvement [4]. These are susceptible to secondary infections. Such infections can precipitate clinical aggravation [3].

There are two types of this condition. The first type appears on one side of the body following Blaschko's lines. The second type is more widespread, with some areas being more severe [2]. The localized form of Darier's disease was first documented in 1906. Since then, various localized forms, including unilateral, linear, segmental, and zosteriform types, have been described in medical literature [4].

We present a case of type I segmental Darier's disease, characterized by lesions restricted to the left side of the trunk and the upper thigh, confirmed by histological evidence.

## 2 Case Presentation

### 2.1 Case History

**FIGURE 1:**
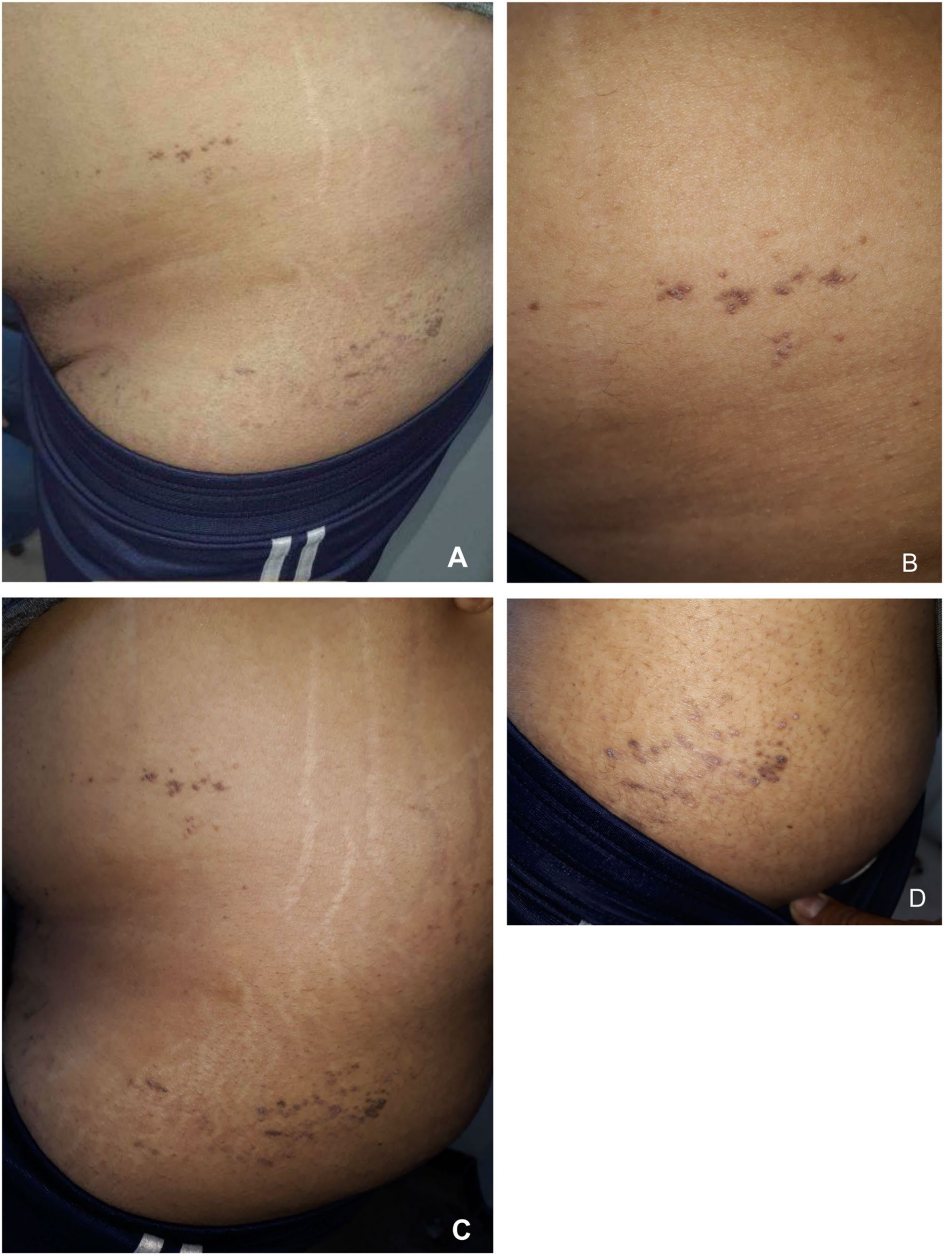
The lesions exhibit a segmental distribution following Blaschko lines, predominantly on the left side of the waist area and the upper left thigh.

A 41‐year‐old male smoker and alcoholic presented with left‐sided small, brown, pruritic, hyperpigmented, keratotic papules for the past 5 months. The lesions exhibit a segmental distribution following Blaschko lines on the left side of the trunk and the upper left thigh (Figure 1).

Additionally, these lesions worsen with increasing environmental temperatures. There was no history of similar skin lesions among any of the family members. There is no previous history of herpes zoster. Additionally, the patient's medical history includes surgical interventions such as appendectomy, fixation of a jaw plate, and tympanic membrane repair.

### 2.2 Investigations, Diagnosis, and Treatment

**FIGURE 2:**
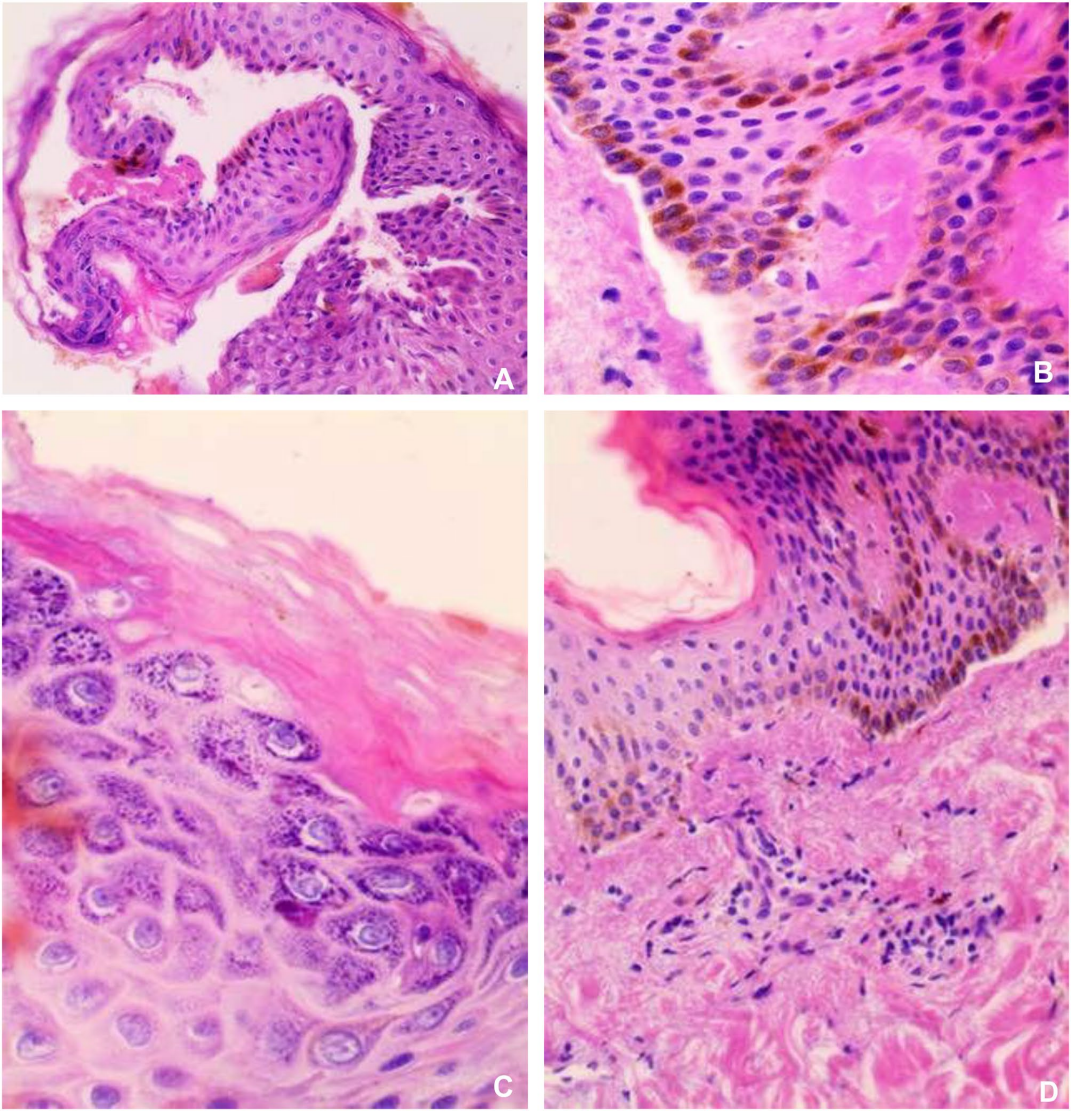
Microscopic description: Parakeratosis, variable epidermal thickness, irregular acantholysis with characteristic dyskeratosis forming corps ronds and grains, suprabasal acantholysis, and clefting with a retained single layer of basal keratinocytes overlying dermal papillae, which appear to project into the acantholytic cavity. Mild mononuclear inflammatory cell infiltrates in the upper dermis.

On dermatological examination, multiple small, brown, pruritic, hyperpigmented, and keratotic papules were observed along Blaschko's lines, unilaterally affecting the left side of the trunk and upper thigh. The lesions, present for the past 5 months, were dry, rough to the touch, and showed no signs of active discharge, erythema, or secondary infection. Examination under natural light revealed a mild shine on the keratotic areas, with the lesions appearing darker at the periphery. Examination of the nails and mucous membranes revealed no abnormalities. The characteristic V‐shaped distal notching, white longitudinal bands or subungual hyperkeratosis typically associated with generalized Darier's disease were absent. The hair and scalp examination also yielded no abnormalities. A detailed skin examination of unaffected areas did not reveal additional lesions. Systemic examination, including cardiovascular, respiratory, gastrointestinal, and neurological evaluations, was unremarkable. Laboratory investigations, including complete blood count, renal and liver function tests, and inflammatory markers, were within normal limits. The patient had no history of immunosuppression or systemic conditions contributing to dermatological manifestations. Histopathological examination of the skin biopsy revealed parakeratosis, variable epidermal thickness, irregular acantholysis with characteristic dyskeratosis forming corp ronds and grains, and suprabasal acantholysis (Figure 2). Based on typical clinical and histopathological findings, he was diagnosed with segmental Darier's disease. Treatment included medium‐strength topical corticosteroids applied twice daily, supplemented by an evening regimen of a topical keratolytic agent, specifically salicylic acid 2% gel, to reduce hyperkeratosis. Additionally, the patient was advised to maintain proper skin hygiene and avoid triggers such as heat and excessive sweating. Retinoids like isotretinoin were not prescribed, as the patient was planning for fertility.

### 2.3 Outcome and Follow‐Up

**FIGURE 3:**
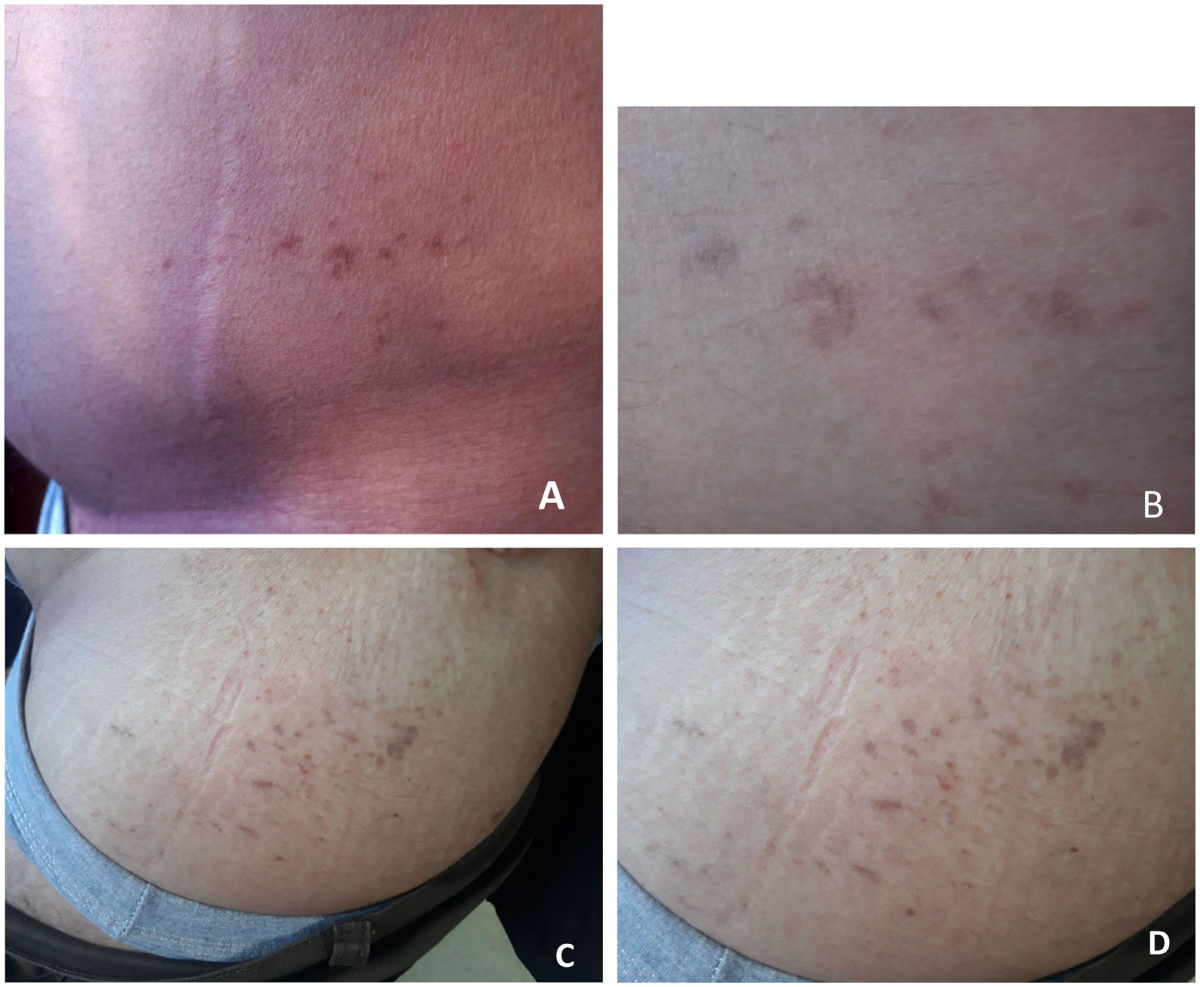
Follow‐up—Cutaneous repair with secondary hyperpigmentation due to inflammation.

A gradual improvement in the patient's condition was observed over 5 months (Figure 3).

## 3 Discussion

Darier's disease, a rare genodermatosis, primarily presents in individuals aged 6–20 years. Clinically, it is characterized by the formation of keratotic papules or plaques in seborrheic regions (trunk, scalp, forehead, and flexures). A V‐shaped notch of the distal nail is also typical for DD. Additionally, nail abnormalities such as erythematous or white longitudinal striations, nail fragility, and subungual keratosis are commonly observed [3, 4]. Patients often report that lesions appear after sun exposure and worsen with heat, sweating, and physical trauma [2]. In our case, the patient's lesions get worse as the temperature rises. The relationship between localized DD and certain epidermal nevi is still controversial. Clinically and histopathologically, localized DD can be indistinguishable from nevi with acantholytic dyskeratosis. It is suggested that epidermal nevi with acantholytic dyskeratosis may be a localized form of DD. Therefore, it has been proposed to rename this condition as segmental DD, believed to result from post‐zygotic mutations [4]. Differential diagnoses may include seborrheic dermatitis, Hailey–Hailey disease, acanthosis nigricans, and epidermodysplasia verruciformis.

However, a definitive diagnosis is established through histopathological examination [5]. In this patient, the localized segmental pattern of skin lesions and specific histopathological changes confirmed the diagnosis. Mosaic Darier disease (DD) is categorized into two types. Type I is characterized by a post‐zygotic mutation that manifests as affected dermatomes along Blaschko's lines surrounded by normal skin. Type II results from a combination of a germline heterozygous mutation and a segmental loss of heterozygosity in the wild‐type allele, leading to localized, exacerbated manifestations of the disease superimposed on the generalized form of DD [3]. The case matches Type I mosaic Darier disease, where the affected skin is in a nonspecific area and the surrounding skin looks normal.

Histopathological analysis is essential for diagnostic confirmation. This is characterized by papillomatosis, hyperkeratosis, and acanthosis, in addition to dyskeratotic keratinocytes that result in acantholysis, grains, and corps ronds [6]. The same findings are found in our case.

There are no standard treatments for type I segmental Darier disease. The main goal is to manage symptoms and improve quality of life. This can be achieved by avoiding triggers, maintaining good hygiene, wearing cotton clothes, using sun protection, and applying medium‐strength topical corticosteroids [1]. Other treatments include topical keratolytic agents like salicylic acid and lactic acid to decrease hyperkeratosis of the lesions. For more severe cases, systemic retinoids including isotretinoin and acitretin are used, bearing in mind their teratogenic potential [7, 8]. In this case, topical corticosteroids and scrubs have yielded positive outcomes, while oral retinoids were abandoned owing to their teratogenicity.

Several cases of segmental Darier's disease have been reported, highlighting diverse presentations, histopathological findings, and treatment outcomes. Most cases involve type I segmental DD with unilateral lesions distributed along Blaschko's lines, confirmed through histopathology showing acantholysis, dyskeratosis, and corps ronds. Treatments commonly include topical corticosteroids, keratolytic agents, or retinoids, with variable responses. Some cases demonstrate spontaneous regression or significant improvement with minimal intervention, while others require prolonged treatment. We summarize previous similar cases and findings (Table 1).

**TABLE 1:** Summarizing findings from previous segmental Darier's Disease cases.

Study	Patient age(years)/sex	Lesion location	Type of segmental DD	Key histopathological findings	Treatment	Outcome
Medeiros et al. 2015 [2]	40/F	Anterior and medial face of right leg	Type I	Ac, Dys, CR, granular layer changes	Topical treatment with adapalene gel 0.3% with urea 20% and salicylic acid 8%	Resolution of lesions after 3 months of treatment
Reese et al. 2005 [3]	48/F	Left abdomen	Unspecified	Ac, Dys, suprabasal clefts, CR	Midpotency topical steroid	Unspecified
Gupta et al. 2013 [4]	35/F	Right inframammary area	Type I	HK, elongation of rete ridges, suprabasal Ac and marked Dys in the form of CR and grains	Unspecified	Regressed spontaneously.
Bidoia et al. 2017 [6]	60/M	Abdomen	Linear	HK, HG, and irregular Ac with suprabasal Ac and Grains in the stratum corneum and Ac	Topical clobetasol 0.05% and acitretin	Good response after 2 months
Suryawanshi, et al. 2017 [7]	35/F	Face, neck, scalp, upper limbs, legs	Generalized, not segmental	HK, Ac, suprabasal clefts, CR	Unspecified	Unspecified
Alsharif et al. 2020 [9]	35/F	Left breast	Type I	HK, Ac, striking suprabasal Ac, and Dys, grains and corps rond	Tretinoin cream 0.01% and hydrocortisone cream 1%	Significant clinical clearance after 1 month
Sanderson et al. 2007 [10]	42/F	Right neck and shoulder	Type 2	Ac with Dys and Dyskeratotic CR and grains	0.1% tretinoin cream and ammonium lactate 12% cream	Cleared in 8 weeks with no recurrence over 10 years
	41/F	Right thigh	Type I	Ac with prominent Dys, Dyskeratotic CR and grains, and a patchy mild superficial perivascular infiltrate in the dermis	Unspecified	Unspecified

Abbreviations: Ac, Acantholysis; CR, Corps Ronds; DD, Darier's Disease; Dys, Dyskeratosis; F, Female; HG, Hypergranulosis; HK, Hyperkeratosis; M, Male.

Furthermore, surgical and non‐surgical procedures may be considered for cases of persistent localized Darier disease unresponsive to standard treatments. Surgical options include excision, dermabrasion, electrosurgery, and laser ablation. Non‐surgical therapies, such as photodynamic therapy, electron beam therapy, and botulinum toxin injections—especially for individuals experiencing pronounced sweating—may also be effective [9]. Nevertheless, despite the potential benefits of surgical interventions for certain patients, the risk of recurrence remains [11].

## 4 Conclusion

In conclusion, this case of type I segmental Darier's disease emphasizes the importance of recognizing its distinctive clinical presentation, particularly the unilateral, Blaschko‐linear distribution, which differentiates it from other skin conditions. Accurate diagnosis through histopathological evaluation is critical for guiding effective treatment; increased awareness of this rare condition can lead to better management strategies and improved quality of life for patients.

## Author Contributions

**Lina Al‐Soufi:** conceptualization, methodology, project administration, supervision, visualization, writing – original draft, writing – review and editing. **Aya Marashli:** investigation, resources, validation, writing – original draft, writing – review and editing. **Hamza Warda:** conceptualization, methodology, validation, writing – original draft, writing – review and editing. **Zuheir Al‐Shehabi:** data curation, project administration, supervision, writing – review and editing.

## Ethics Statement

The authors have nothing to report.

## Consent

Written informed consent was obtained from the patient to publish this report in accordance with the journal's patient consent policy.

## Conflicts of Interest

The authors declare no conflicts of interest.

## Data Availability

The data that support the findings of this study are available from the corresponding author upon reasonable request.
